# Plasma membrane H^+^-ATPases promote TORC1 activation in plant suspension cells

**DOI:** 10.1016/j.isci.2022.104238

**Published:** 2022-04-11

**Authors:** Cecilia Primo, Catherine Navarre, François Chaumont, Bruno André

**Affiliations:** 1 Molecular Physiology of the Cell, Université Libre de Bruxelles (ULB), Biopark, B-6041 Gosselies, Belgium; 2 Louvain Institute of Biomolecular Science and Technology, UCLouvain, B-1348 Louvain-la-Neuve, Belgium

**Keywords:** Biological sciences, Plant biology, Molecular plant pathology

## Abstract

The TORC1 (Target of Rapamycin Complex 1) kinase complex plays a pivotal role in controlling cell growth in probably all eukaryotic species. The signals and mechanisms regulating TORC1 have been intensely studied in mammals but those of fungi and plants are much less known. We have previously reported that the yeast plasma membrane H^+^-ATPase Pma1 promotes TORC1 activation when stimulated by cytosolic acidification or nutrient-uptake-coupled H^+^ influx. Furthermore, a homologous plant H^+^-ATPase can substitute for yeast Pma1 to promote this H^+^-elicited TORC1 activation. We here report that TORC1 activity in *Nicotiana tabacum* BY-2 cells is also strongly influenced by the activity of plasma membrane H^+^-ATPases. In particular, stimulation of H^+^-ATPases by fusicoccin activates TORC1, and this response is also observed in cells transferred to a nutrient-free and auxin-free medium. Our results suggest that plant H^+^-ATPases, known to be regulated by practically all factors controlling cell growth, contribute to TOR signaling.

## Highlights

• Isolation of a tobacco BY-2 cell line suitable for analyzing TOR signaling
• Activation of plasma membrane H^+^-ATPases in BY-2 suspension cells elicits TOR signaling
• TOR signaling upon H^+^-ATPase activation also occurs in the absence of nutrients and auxin

## Graphical abstract

**Figure:**
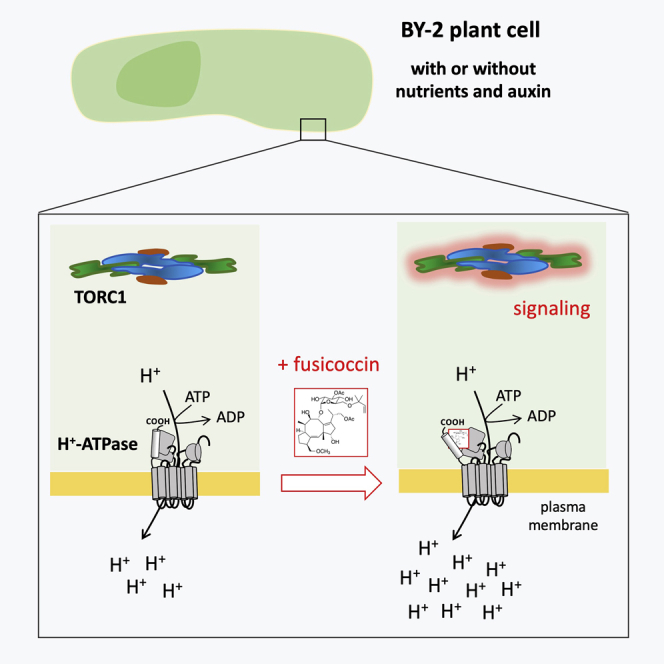

## Introduction

The TORC1 (Target of Rapamycin Complex 1) kinase complex (Heitman et al., 1991; Loewith et al., 2002) is now recognized as a central hub integrating multiple environmental signals to control cell growth in probably all eukaryotic species (Brunkard, 2020; Kim and Guan, 2019; Liu and Sabatini, 2020). These signals include nutrient availability, growth hormone detection, the energetic status of the cell, and stress conditions. The mechanisms controlling TORC1 activity in response to these input cues have been best studied in mammals, where links with several pathologies have been established (Liu and Sabatini, 2020; Mossmann et al., 2018). How TORC1 is regulated in fungi and plants, however, has been much less characterized.

A common way to study TORC1 control by nutrients is to cause TORC1 inactivation by starving cells for a limited time and then to replenish them with the limiting nutrient, causing TORC1 reactivation. Studies applying this methodology to human cells have led to the discovery of specific cytosolic amino acid sensor proteins capable of inhibiting mTORC1 when intracellular leucine, arginine, or S-adenosyl-methionine become scarce (Wolfson and Sabatini, 2017). These amino acid sensors act through GATOR complexes controlling a heterodimer of RagA/B and RagC/D small GTPases that importantly contribute to activation of mTORC1 by recruiting it to the lysosome (González and Hall, 2017; Kim and Guan, 2019; Liu and Sabatini, 2020; Nicastro et al., 2017). In yeast and plant cells, TORC1 also shows low activity under nitrogen starvation conditions and is reactivated upon uptake of external nitrogenous compounds such as amino acids or NH_4_^+^ (Bonfils et al., 2012; Crespo et al., 2002; Liu et al., 2021; Stracka et al., 2014; Urban et al., 2007). In these species, however, the nitrogen sensing mechanisms promoting this TORC1 reactivation are much less well characterized (Dobrenel et al., 2016; Nicastro et al., 2017; Wu et al., 2019), and the cytosolic amino acid sensors described in human cells do not seem conserved (Brunkard, 2020; Wolfson and Sabatini, 2017).

A common feature of fungal and plant cells, distinguishing them from animal cells, is that their plasma membrane is energized by highly conserved H^+^-ATPases of the P-type ATPase family (Duby and Boutry, 2009; Falhof et al., 2016; Kane, 2016; Palmgren and Morsomme, 2018; Portillo, 2000; Serrano, 1993). These proteins establish an H^+^ gradient driving active uptake of ions and nutrients by multiple transporters coupled to H^+^ influx. This H^+^ gradient is also a main component of the membrane potential. In a previous study, we found that in yeast cells grown under nitrogen limitation, initial TORC1 reactivation upon amino acid uptake is not because of accumulation of the amino acid itself but to H^+^ influx coupled to amino acid transport (Saliba et al., 2018). As starvation for a specific nutrient typically leads to abundant synthesis of high-affinity H^+^-symporters able to assimilate replenishing compounds, we proposed that H^+^ influx via such transporters, upon nutrient transport, provides a general signal for rapid and transient TORC1 reactivation upon exit from starvation. This initial TORC1 activation is followed by a more sustained activation dependent on proper assimilation of the internalized nutrient (Stracka et al., 2014). Further analysis revealed that the H^+^ influx is required but not sufficient to promote TORC1 activation. Specifically, the yeast plasma membrane H^+^-ATPase, Pma1, known to be stimulated when the cytosol becomes acidic (Kane, 2016; Portillo, 2000), plays an essential role in nutrient-uptake-elicited TORC1 activation. This is supported by the observation that in yeast cells where Pma1 is replaced with the tobacco (*Nicotiana plumbaginifolia*) PMA4 H^+^-ATPase truncated of its cytosolic C-terminal tail, equivalent amino acid uptake coupled to H^+^ influx fails to support TORC1 activation (Saliba et al., 2018). Furthermore, TORC1 is also stimulated in Pma1-expressing cells treated with a V-ATPase complex inhibitor or protonophore (conditions causing cytosol acidification), but not in cells where Pma1 is replaced with the C-terminally truncated plant PMA4 H^+^-ATPase (Saliba et al., 2018). Further analysis revealed that when the endogenous Pma1 is replaced with the full-length version of PMA2, another *N. plumbaginifolia* H^+^-ATPase isoform, yeast TORC1 is efficiently activated upon transport-coupled H^+^ influx or upon a H^+^ increase in the cytosol. This activation of TORC1 is lost when PMA2 is truncated of its C-terminal tail, and this effect does not correlate with reduced activity of the H^+^-ATPase (Saliba et al., 2021). This illustrates that at least some plant H^+^-ATPases, via their C-terminal tails, can support activation of yeast TORC1 upon an H^+^ increase. The molecular mechanisms underlying H^+^-elicited activation of yeast TORC1 via endogenous or heterologous plant H^+^-ATPases remain uncharacterized.

Plant plasma membrane H^+^-ATPases, existing as multiple isoforms, play a crucial role in controlling plant growth. This function is intimately linked to the autoinhibitory C-terminal tail of these H^+^ pumps. The regulatory effect of this cytosolic region is modulated by phosphorylation of several key residues (Duby and Boutry, 2009; Falhof et al., 2016; Haruta et al., 2015). This phosphorylation responds to multiple growth-impacting environmental signals, such as blue light, phytohormones, and extracellular peptides (Falhof et al., 2016). When fully active, plant H^+^-ATPases are typically phosphorylated on the penultimate Thr. This creates a binding site for 14-3-3 proteins, which stabilize the active state of H^+^-ATPases where autoinhibition by the C-terminus is neutralized (Duby and Boutry, 2009; Falhof et al., 2016; Haruta et al., 2015). Plant H^+^-ATPases are phosphorylated on the penultimate Thr also when expressed in yeast, and this promotes both their association with yeast endogenous 14-3-3s and their full activation (Fuglsang et al., 1999; Maudoux et al., 2000). Interestingly, substitutions in the C-terminal tail of tobacco PMA2 shown to impair interaction with 14-3-3s (Maudoux et al., 2000) also reduce the ability of this plant H^+^-ATPase to support TORC1 activation in yeast, and a similar effect is observed when expression of yeast endogenous 14-3-3s is reduced (Saliba et al., 2021). Association of H^+^-ATPases with 14-3-3s also favors formation of a heterododecameric structure consisting of six ATPases and six 14-3-3s (Kanczewska et al., 2005; Ottmann et al., 2007). Fusicoccin, a fungal toxin triggering strong activation of plant H^+^-ATPases (Marra et al., 2021), acts by intercalating between the C-terminal tails of the H^+^ pumps and 14-3-3s, thereby irreversibly stabilizing the 14-3-3/H^+^-ATPase complex even when the extreme C-terminus of the H^+^-ATPase is not phosphorylated (Baunsgaard et al., 1998; Piotrowski et al., 1998; Würtele et al., 2003). The C-terminal tail of the yeast Pma1 H^+^-ATPase, although shorter than equivalent regions in plant H^+^-ATPases, also functions as an autoinhibitory domain whose effect is likewise modulated by phosphorylation of several key residues (Palmgren and Morsomme, 2018; Portillo, 2000). It does not seem, however, to bind 14-3-3s (Saliba et al., 2021). Several fungal H^+^-ATPases are nevertheless reported to associate into hexamers (Chadwick et al., 1987; Ruiz-Granados et al., 2019), whose structure has recently been solved (Heit et al., 2021; Zhao et al., 2021).

Yeast and plant H^+^-ATPases thus share remarkable structural, functional, and regulatory properties, and this similarity extends to their ability to support H^+^-mediated TORC1 activation in yeast cells. Yet, whether H^+^-ATPases also influence TORC1 activity in plant cells has not been investigated to date. Here, we first describe conditions for assessing TORC1 activity in a *Nicotiana tabacum* Bright Yellow-2 (BY-2) cell line adapted for this purpose. We then show that plasma membrane H^+^-ATPases strongly influence TORC1 activity and do so in a manner that goes beyond their role in driving active nutrient uptake.

## Results

### Isolation of a tobacco BY-2 cell line suitable for studying TOR signaling

**Figure 1:**
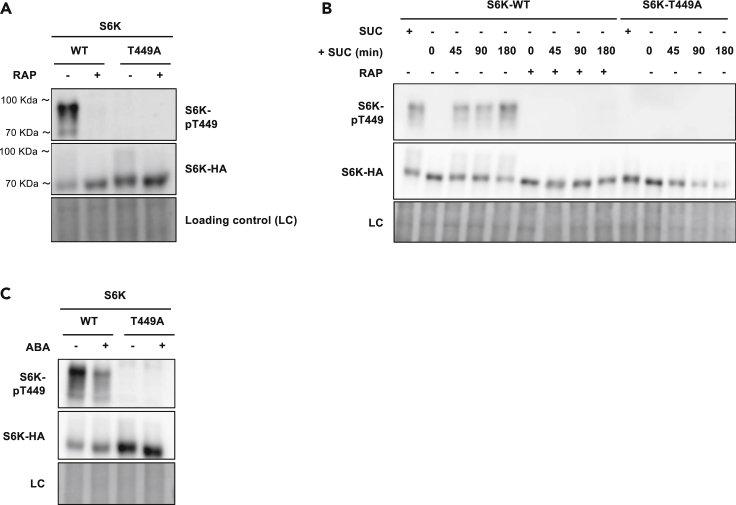
Isolation of a tobacco BY-2 cell line suitable for studying TOR signaling (A) Representative immunoblot illustrating the effect of rapamycin on S6K1 phosphorylation in BY-2 cells. Cells expressing S6K1-HA (line S6KHA-11) or S6K1-T449A-HA were grown for three days in sucrose-containing MS medium, transferred to fresh MS medium for one more day, and treated for 1 h with 10 μM rapamycin (RAP) (+) or the vehicle DMSO (−). Protein extracts prepared from cell samples were immunoblotted with anti-pT449-S6K1 (upper panel) and anti-HA (middle panel) antibodies. The lower panel corresponds to the direct blue-stained filter for detection of total proteins used as a loading control (LC). (B) Influence of sucrose on S6K1 phosphorylation in BY-2 cells. Cell lines as in A (SUC +) were grown for three days in sucrose-containing MS medium and starved of sucrose for 24 h. Sucrose (3%) was then added again to the cells pretreated or not with rapamycin (10 μM) for 2 h, and cell samples were collected at the indicated times. Protein extracts were prepared and immunoblotted as in A. (C) Influence of abscisic acid on S6K1 phosphorylation in BY-2 cells. Lines and growth conditions as in A, except that cells were treated for 3 h with 50 μM abscisic acid (ABA) (+) or the vehicle ethanol (−). Protein extracts were immunoblotted as in A.

BY-2 cells from *Nicotiana tabacum* (Nagata et al., 1992) have proved particularly useful in studying the regulation of plant plasma membrane H^+^-ATPases (Bobik et al., 2010; Kanczewska et al., 2005). Their growth in defined liquid medium can be readily monitored, and homogenous cell samples can be retrieved from the cultures in order to study cellular responses induced by specific treatments or changes in medium composition. Therefore, we chose this plant cell line to investigate potential links between the activity of H^+^-ATPases and TOR signaling. In a recent study, it was shown that TOR activity can be assessed in *Arabidopsis thaliana* suspension cells by detecting increased phosphorylation of residue Thr449 in a stably overexpressed S6K1 kinase (a direct TORC1 target commonly used as readout of TORC1 activity) tagged at its C-terminus with a triple HA (Van Leene et al., 2019). We inserted the same construct into BY-2 cells, thus isolating three independent cell lines stably expressing S6K1-3HA at roughly the same level (Figure S1A). Cells of one of these BY-2 cell lines (S6KHA-11) were grown in liquid Murashige & Skoog (MS)-sucrose medium and collected during the phase of optimal growth. Proteins were extracted and probed with a polyclonal antibody directed against a phospho-T449-containing peptide from the *A. thaliana* S6K1 protein (Figure 1A). The immunoblot revealed several bands well above the main S6K1 signal detected with an anti-HA antibody. These upper bands correspond to *A. thaliana* S6K1 forms phosphorylated on several residues including T449, as they were undetectable in BY-2 cells stably expressing the same HA-tagged S6K1 construct with the T449A substitution (Figure 1A). Furthermore, these phosphorylated S6K1 forms do not correspond to the endogenous S6K because they were not detected in untransformed BY-2 cells (Figure S1A). Finally, that this S6K1 phosphorylation is TORC1 dependent was confirmed by its loss of detection in cells treated for 1 h with the TORC1 inhibitors rapamycin (10 μM) (Figure 1A) or AZD8055 (1 μM) (Figure S1B).

The TOR kinase is active in sugar-fed plant root cells and inactive under sugar starvation (Xiong et al., 2013). We similarly observed a strong decrease in S6K1 phosphorylation in BY-2 cells transferred for 24 h to a sucrose-free medium (Figure 1B). Sucrose repletion rapidly restored S6K1 phosphorylation unless the cells were pretreated with rapamycin (Figure 1B). Equivalent TORC1 control by sucrose was observed in the other two isolated BY-2 cell lines (Figure S1B).

The phytohormone abscisic acid (ABA) plays a central role in integrating various stress signals and promoting stress tolerance at the expense of growth. Recent works report that ABA inhibits TORC1 activity via the evolutionarily conserved energy-sensing SNF1-related protein kinases one and 2 (SnRK1 and -2) (Belda-Palazón et al., 2020; Wang et al., 2018). In BY-2 cells incubated with ABA, we likewise observed markedly reduced S6K1 phosphorylation (Figure 1C).

In conclusion, we have isolated BY-2 cell lines suitable for analyzing TORC1 activity by monitoring S6K1-T449 phosphorylation. Furthermore, the activity of TORC1 in these cells is sensitive to TORC1 inhibitors and responds as expected to sucrose and ABA.

### Inhibition of plasma membrane H^+^-ATPases causes rapid TOR inactivation

**Figure 2:**
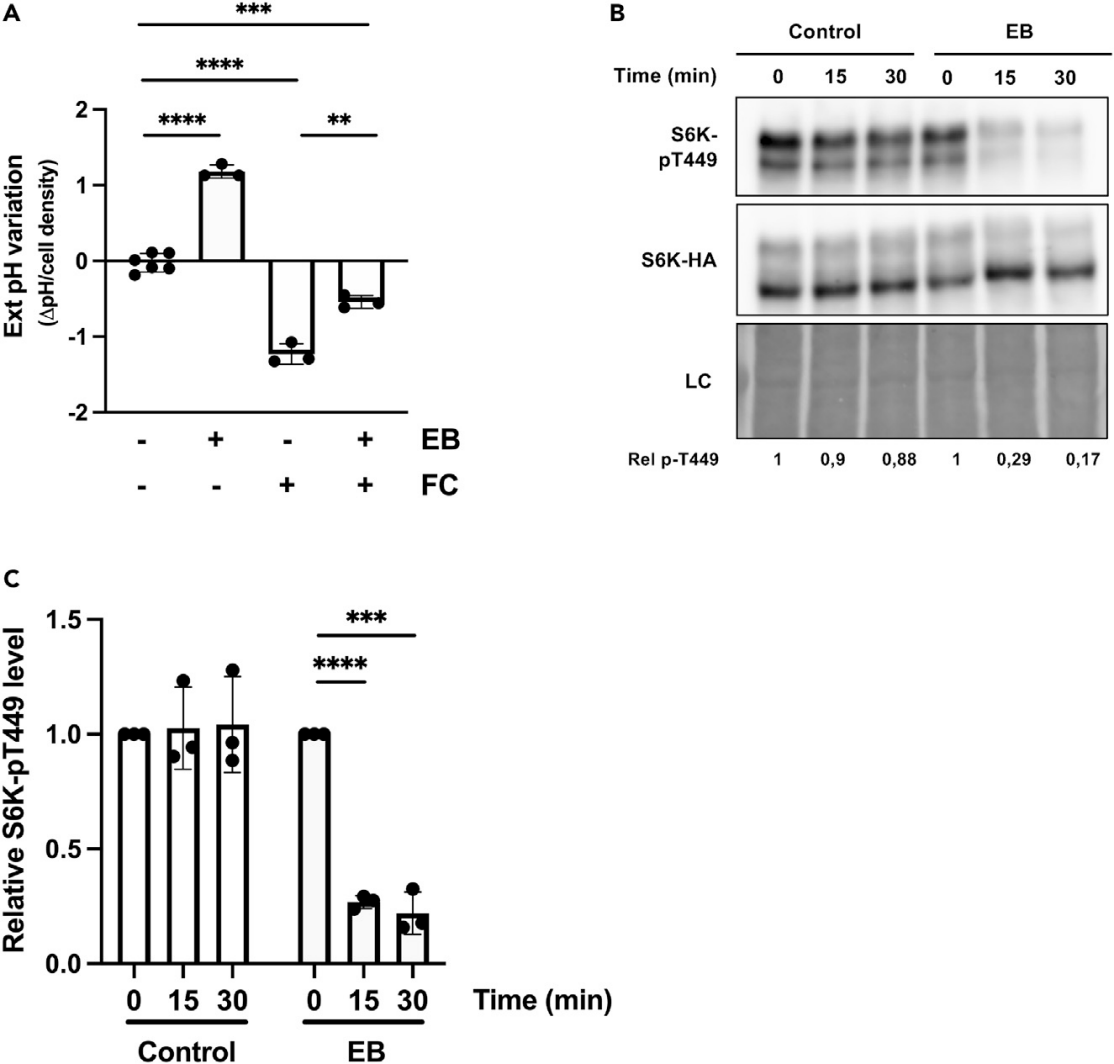
Inhibition of plasma membrane H^+^-ATPases results in rapid TOR inactivation (A) External pH variation after cell treatment with erythrosine B (EB) or fusicoccin (FC). Cells of BY-2 line S6KHA-11 grown in MS medium for three days and transferred to fresh medium for one more day were treated (+) or not (−) for 15 min with EB (30 μM) or FC (2.5 μM) or pretreated for 15 min with EB and then treated with FC. Bars represent means ± standard deviation (SD), n ≥ 3. Asterisks indicate significant differences assessed with the two-tailed *t*-test (∗∗p ≤ 0.01, ∗∗∗p ≤ 0.001). (B) Representative immunoblot illustrating the effect of EB on S6K1 phosphorylation. Cells as in A were treated for 15 and 30 min with EB (30 μM) or not treated (Control). Protein extracts were immunoblotted with anti-pT449-S6K1 (upper panel) and anti-HA (middle panel) antibodies. The lower panel corresponds to a loading control (LC) as in Figure 1. Rel p-T449 corresponds to relative S6K1-pT449 phosphorylation as detailed in C. (C) Quantification, from independent experiments performed as in (B), of the influence of EB on S6K1 phosphorylation. Values represent S6K1-pT449 vs. S6K1-HA signal intensity ratios normalized using time ‘0 min’ as the reference (set at 1), n = 3. Bars represent means ± SD Asterisks indicate significant differences assessed with a two-tailed *t*-test (∗∗∗p ≤ 0.001).

We have recently reported that the yeast plasma membrane H^+^-ATPase Pma1 plays an essential role in initial TORC1 reactivation upon active nutrient uptake into starved cells (Saliba et al., 2018). Furthermore, this Pma1-dependent TORC1 activation can be achieved by the closely related PMA2 H^+^-ATPase from the plant *Nicotiana plumbaginifolia* but not by an equally active mutant derivative truncated of its inhibitory C-terminal tail (Saliba et al., 2021). On the other hand, the activity of plant plasma membrane H^+^-ATPases is known to be tightly regulated according to multiple environmental cues (Falhof et al., 2016), many of which (e.g., sucrose, auxin, ABA) also influence TOR activity (Wu et al., 2019). We thus used BY-2 cells, mainly those of the previously described S6KHA-11 line, as a tool to further investigate potential links between the activity of H^+^-ATPases and TOR signaling. As a first step, we treated cells with erythrosine B, a fluorescein derivative shown to inhibit the activity of plant H^+^-ATPases (Cocucci and Marré, 1986). In support of the view that H^+^-ATPases are inhibited *in vivo* in treated BY-2 cells, addition of erythrosine B for 15 min caused reproducible alkalinization of the growth medium (Figure 2A). Furthermore, treating the same BY-2 cells with fusicoccin, a fungal toxin well established to stimulate the activity of plasma membrane H^+^-ATPases (Marra et al., 2021), caused the opposite effect—acidification of the medium—that was significantly counteracted by preincubating the cells with erythrosine B (Figure 2A). We then compared S6K1 phosphorylation in cells treated or not with erythrosine B. Interestingly, S6K1 phosphorylation was markedly reduced already 15 min after the treatment (Figures 2B and 2C). Although these observations indicate that inhibition of H^+^-ATPases by erythrosine B causes TORC1 inactivation, one should note that this compound likely perturbs the activity of other ATPases. For instance, the activity of plant Ca^2+^-ATPases is reported to be sensitive to erythrosine B (Evans and Williams, 1998). This prompted us to incubate BY-2 cells also with vanadate, a general P-type ATPase inhibitor often used to inactivate plasma membrane H^+^-ATPases (Palmgren and Morsomme, 2018). This treatment, which expectedly caused alkalinization of the medium, also reduced S6K1 phosphorylation (Figures S2A–S2C). When used at higher concentrations, vanadate seemed detrimental because much less proteins were reproducibly extracted from cell samples (Figure S2D).

TORC1 is thus rapidly inhibited in BY-2 cells treated with two different inhibitors of plasma membrane H^+^-ATPases, suggesting that sustained TOR signaling requires that these pumps be active. Yet, as the two inhibitors used do not specifically target H^+^-ATPases, we cannot rule out the possibility that the observed inhibition of TORC1 was caused, at least partially, by inhibition of other ATPases that might contribute to maintaining high TORC1 activity.

### Stimulation by fusicoccin of the activity of plasma membrane H^+^-ATPases activates TOR

**Figure 3:**
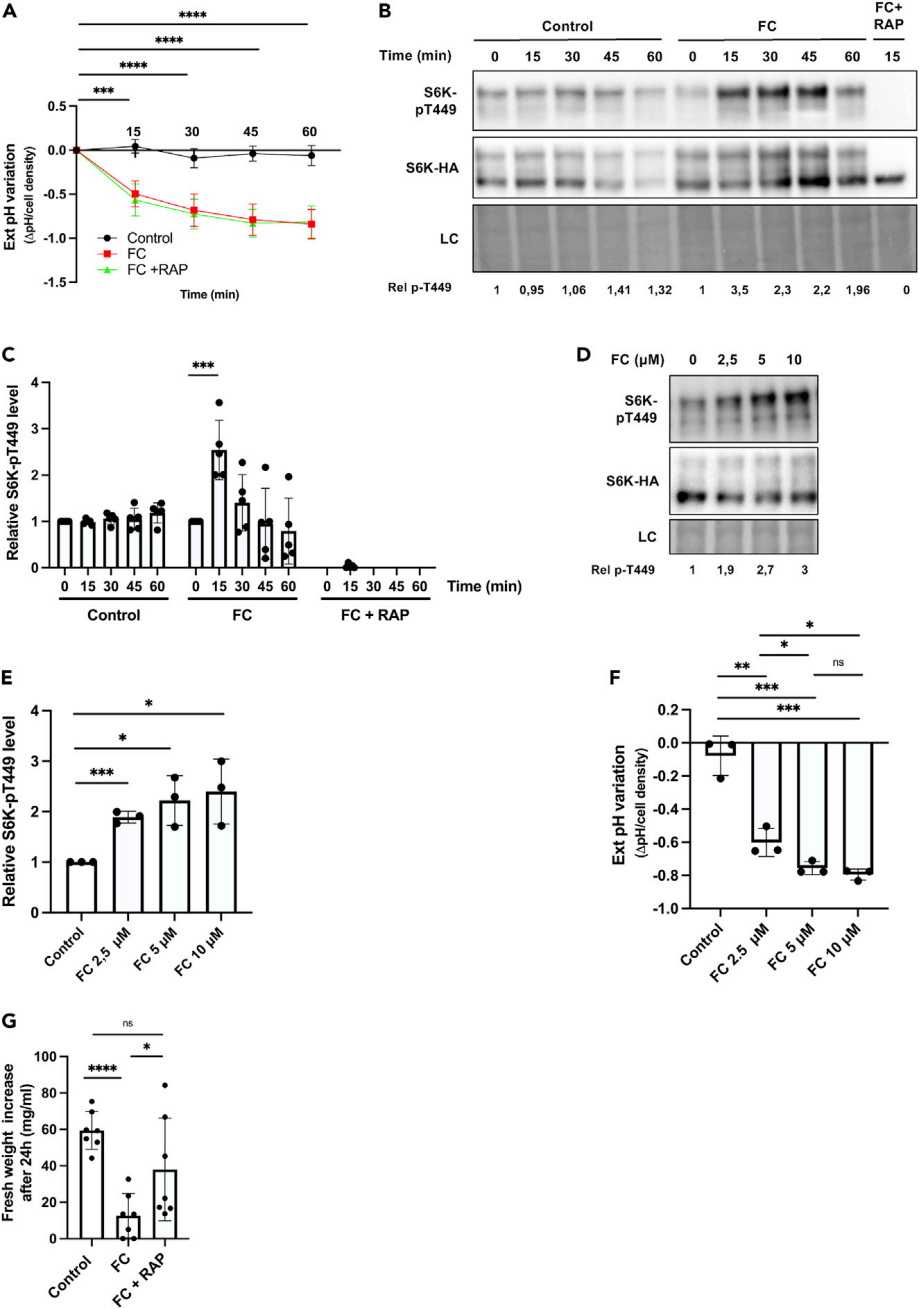
Stimulation of plasma membrane H^+^-ATPase activity by fusicoccin transiently activates TOR (A) External pH variation after cell treatment with fusicoccin (FC). Cells of the BY-2 line S6KHA-11 were grown in MS medium for 3 days, transferred to fresh MS medium, cultivated for three more days, and treated with FC (2.5 μM) or the vehicle ethanol (Control) or with FC after pretreatment for 2 h with rapamycin (10 μM) (FC + RAP). Values are means ± SD, n = 5. Asterisks indicate significant differences vs. untreated cells, for both FC and FC + RAP conditions, as assessed with the two-tailed *t*-test (∗∗∗p ≤ 0.001). (B) Representative immunoblot illustrating the influence of FC on S6K1 phosphorylation. Cells as in A were treated for the indicated times with FC (2.5 μM) or the vehicle ethanol (Control). The effect of a 15-min treatment with FC was also analyzed in cells pretreated for 2 h with rapamycin (10 μM). Protein extracts were immunoblotted with anti-pT449-S6K1 (upper panel) and anti-HA (middle panel) antibodies. The lower panel corresponds to a loading control (LC), as in Figure 1. Rel p-T449 corresponds to relative S6K1-pT449 phosphorylation as detailed in C. (C) Quantification, from independent experiments performed as in (B), of the influence of FC on S6K1 phosphorylation. Values represent S6K1-pT449 vs. S6K1-HA signal intensity ratios normalized using time ‘0 min’ as the reference (set at 1), n = 5. Bars represent means ± SD Asterisks indicate significant differences assessed with the two-tailed *t*-test (∗∗∗p ≤ 0.001). (D) Representative immunoblot illustrating the influence of increasing concentrations of FC on S6K1 phosphorylation. Cells as in A were treated for 15 min with different concentrations of FC or with the vehicle ethanol (FC 0 μM). Protein extracts prepared from cell samples were immunoblotted as in (B). Rel p-T449 corresponds to relative S6K1-pT449 phosphorylation as detailed in E. (E) Quantification, from independent experiments performed as in (D), of the influence of FC added at various concentrations. Values were calculated as in (C) and bars represent means ± SD (n = 3). Asterisks indicate significant differences assessed with the two-tailed *t*-test (∗p ≤ 0.05, ∗∗∗p ≤ 0.001). (F) External pH variation after cell treatment with increasing concentrations of FC. Cells and conditions as in (D). Bars represent means ± SD, n = 3. Asterisks indicate significant differences assessed with the two-tailed *t*-test (∗p ≤ 0.05, ∗∗p ≤ 0.01, ∗∗∗p ≤ 0.001, ns denotes not significant). (G) Influence of FC on growth. Cells of the BY-2 line S6KHA-11 were grown in MS medium for three days and transferred to a fresh MS medium for three more days. The cultures were then treated with FC (2.5 μM) in the presence or absence of rapamycin (10 μM) and after 24 h the increase in fresh weight (mg/mL) was measured. Control is untreated cells. Bars represent means ± SD (n = 7). Asterisks indicate significant differences assessed with the two-tailed *t*-test (∗p ≤ 0.05, ∗∗∗p ≤ 0.001, ns denotes for not significant).

The H^+^ gradient established by plasma membrane H^+^-ATPases drives active uptake of multiple nutrients, including sucrose. It thus came as no surprise that inhibition of the H^+^ pumps caused TORC1 inhibition. Yet, this observation is also compatible with the view that H^+^ ATPases in BY-2 cells additionally play a more direct role in controlling TOR signaling as recently proposed for the homologous yeast Pma1 H^+^-ATPase (Saliba et al., 2018) and for the *N. plumbaginifolia* PMA2 H^+^-ATPase expressed in yeast (Saliba et al., 2021). To further assess this view, we analyzed TORC1 activity in BY-2 cells treated with fusicoccin (FC). This extensively studied fungal phytotoxin (Marra et al., 2021) is well-known to directly interact with and provoke hyperactivation of plasma membrane H^+^-ATPases *in vivo* (Palmgren and Morsomme, 2018). To assess the influence of FC on TORC1 activity, a freshly inoculated culture of BY-2 cells was first incubated for three days. In cells grown under these conditions, TORC1 activity is relatively low, likely because nutrients become limited. Addition of FC caused rapid acidification of the medium, indicating that the activity of H^+^-ATPases was stimulated as expected (Figure 3A). Interestingly, immunoblot analysis of cell extracts revealed a net increase in S6K1 phosphorylation upon FC addition, followed by a drop to the initial level (Figures 3B and 3C). This transient S6K1 phosphorylation was not observed in cells treated with the vehicle control. Nor was it detected in FC-treated cells preincubated with rapamycin (Figures 3B and 3C), which did not interfere with FC-induced H^+^-ATPase stimulation as judged by acidification of the medium (Figure 3A). Furthermore, this FC-elicited increase in S6K1 phosphorylation seemed more pronounced when FC was added at higher concentration, and this correlated with significantly increased acidification of the medium (Figures 3D–3F). These results show that stimulating the activity of H^+^-ATPases with FC promotes TORC1 activation in BY-2 cells. In addition, this activation occurs only transiently, although the H^+^-ATPases seem to remain hyperactive. This could be because of some natural feedback inhibition of TORC1. Alternatively, cells in which TORC1 has been artificially overactivated by FC treatment might progressively enter a stress state associated with triggering of TORC1 inhibition mechanisms. In support of the view that long-term FC treatment is stressful for cells, we found that incubating BY-2 cells for 24 h with FC strongly reduced the biomass increase (Figure 3G). Interestingly, simultaneous treatment of these cells with rapamycin partially compensated for this toxic effect of FC (Figure 3G) and similar results were obtained with the other two BY-2 cell lines isolated in this study (Figure S3A). This suggests that the detrimental effect of FC on BY-2 cell growth is caused, at least partially, by excessive TORC1 activation. Of note, rapamycin added alone at the concentration used in this experiment did not significantly interfere with growth (Figure S3B).

### H^+^-ATPase stimulation by fusicoccin activates TOR independently of external nutrient uptake

**Figure 4:**
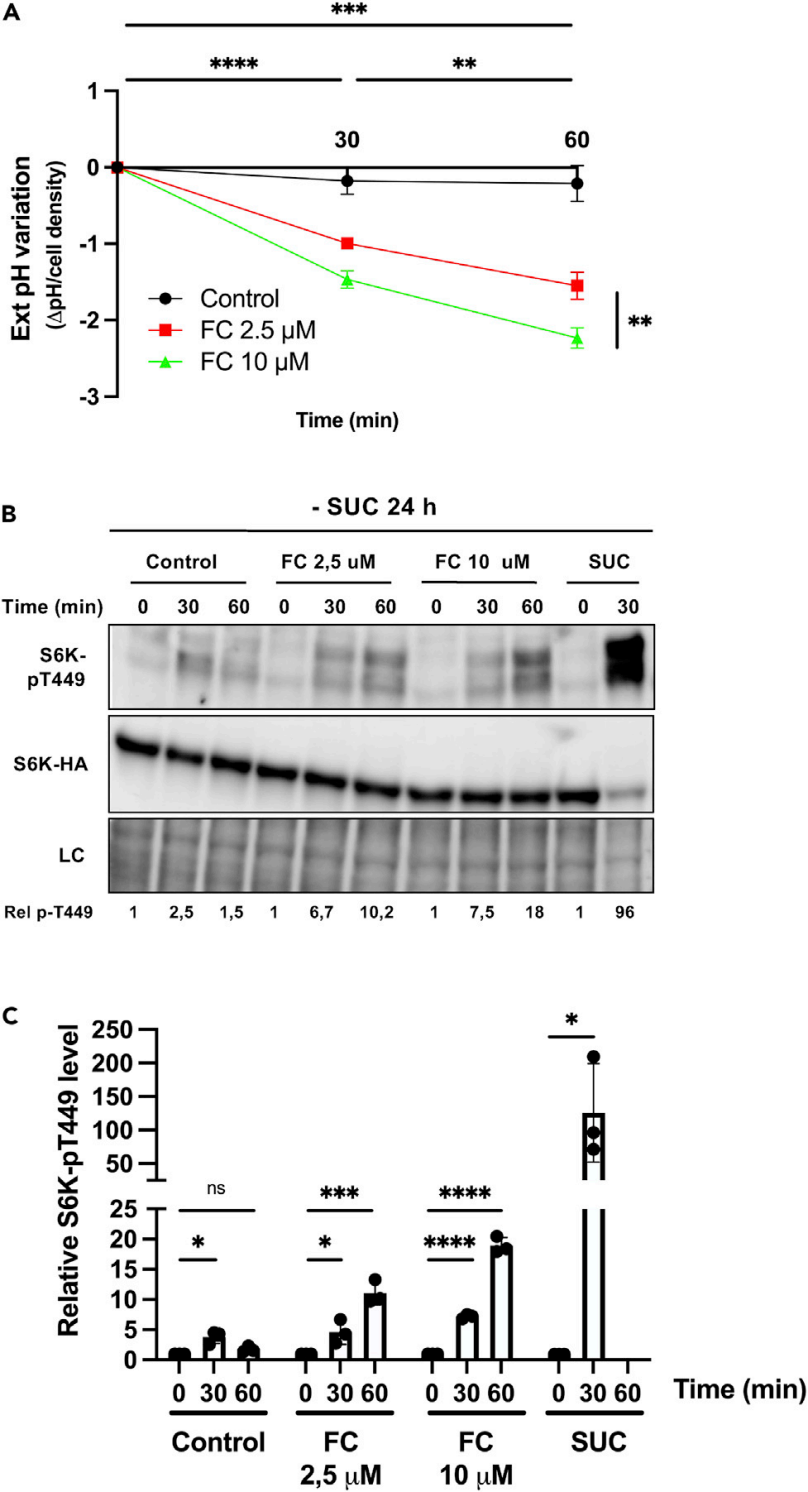
Fusicoccin activates TOR in sucrose-starved BY-2 cells (A) External pH variation after treating sucrose-starved cells with fusicoccin (FC). BY-2 cells of the line S6KHA-11, grown in sucrose-containing MS medium for three days and then starved of sucrose for 24 h, were treated with FC (2.5 or 10 μM) or the vehicle ethanol (Control). Values are means ± SD, n = 3. Asterisks for comparisons between time points indicate significant differences vs. untreated cells for both FC concentrations, as assessed with the two-tailed *t*-test (∗∗p ≤ 0.01,∗∗∗p ≤ 0.001, ∗∗∗∗p ≤ 0.0001). (B) Representative immunoblot illustrating the influence of FC on S6K1 phosphorylation in sucrose-starved cells. Cells as in A were treated for 30 or 60 min with FC (2.5 or 10 μM) or with the vehicle ethanol (Control). As a positive control of S6K1 phosphorylation, sucrose (SUC) was added for 30 min to the 24-h starved cells. Protein extracts from cell samples were immunoblotted with anti-pT449-S6K1 (upper panel) and anti-HA (middle panel) antibodies. The lower panel corresponds to a loading control (LC), as in Figure 1. Rel p-T449 corresponds to relative S6K1-pT449 phosphorylation as detailed in C. (C) Quantification, from independent experiments performed as in (B), of the influence of FC on S6K1 phosphorylation in sucrose-starved cells. Values represent S6K1-pT449 vs. S6K1-HA signal intensity ratios normalized using time ‘0 min’ as the reference (set at 1), n = 3. Bars represent means ± SD. Asterisks indicate significant differences assessed with the two-tailed *t*-test (*∗*p ≤ 0.05, ∗∗∗p ≤ 0.001, ns denotes not significant).

A mechanism that might account for the above-described TORC1 activation in FC-treated BY-2 cells is as follows: stimulating the activity of H^+^-ATPases could boost H^+^-coupled uptake of nutrients; for instance, sucrose via secondary active transporters. To assess this view, BY-2 cells cultured for three days were transferred for 24 h to the same medium except that it lacked sucrose. After this incubation, FC was supplied at two different concentrations (2.5 and 10 μM). This resulted in acidification of the external medium to different degrees (Figure 4A), indicating that H^+^-ATPases had been activated by FC despite the carbon-starved state of the cells. Immunoblot analysis of cell extracts showed that FC addition under these conditions also caused a significant increase in S6K1 phosphorylation, particularly visible when FC was added at the higher concentration (Figures 4B and 4C). Although significant, this S6K1 phosphorylation was of lesser amplitude than that detected 30 min after sucrose re-addition (Figures 4B and 4C). These observations indicate that stimulating the activity of H^+^-ATPases with FC triggers sucrose-uptake-independent activation of TORC1.

**Figure 5:**
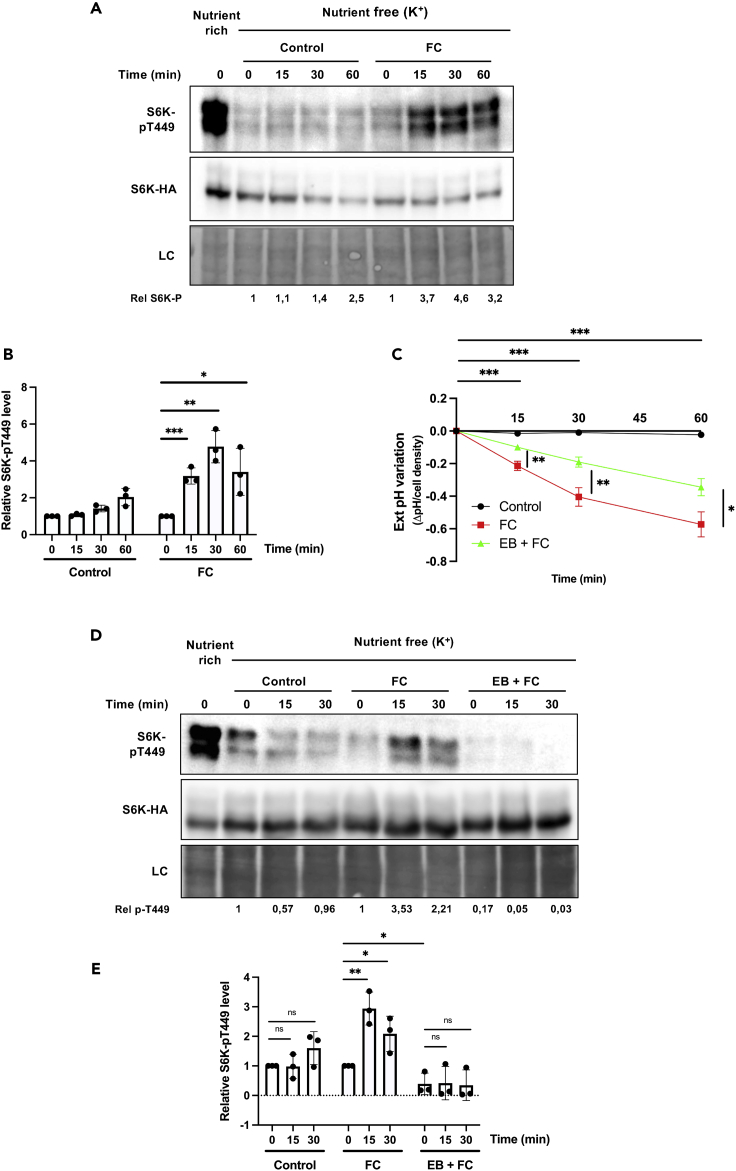
Stimulation of plasma membrane H^+^-ATPase activity by fusicoccin transiently activates TOR in nutrient-free conditions (A) Representative immunoblot illustrating the influence of fusicoccin (FC) on S6K1 phosphorylation under nutrient-free conditions. BY-2 cells of S6KHA-11 line grown in MS medium for three days and transferred for three more days to fresh MS medium were collected and transferred for 2 h to buffer referred to as “Nutrient free (K^+^)” (5 mM MES, pH adjusted to 5.8 with KOH). The cells were then treated with FC (5 μM) or the vehicle ethanol (Control). Protein extracts from cell samples were immunoblotted with anti-pT449-S6K1 (upper panel) and anti-HA (middle panel) antibodies. The lower panel corresponds to a loading control (LC), as in Figure 1. As a positive control of S6K1 phosphorylation, a sample was collected before the shift to MES buffer (Nutrient rich, time 0). Rel p-T449 corresponds to relative S6K1-pT449 phosphorylation as detailed in B. (B) Quantification, from independent experiments performed as in (A), of the influence of FC on S6K1 phosphorylation under nutrient-free conditions. Values represent S6K1-pT449 vs. S6K1-HA signal intensity ratios normalized using time ‘0 min’ as the reference (set at 1), n = 3. Bars represent means ± SD Asterisks indicate significant differences assessed with the two-tailed *t*-test (∗p ≤ 0.05, ∗∗p ≤ 0.01, ∗∗∗p ≤ 0.001). (C) External pH variation after treating cells with FC or FC and EB under nutrient-free conditions. Cells and conditions as in (A). Values are means ± SD, n = 3. Asterisks indicate significant differences assessed with the two-tailed *t*-test (∗p ≤ 0.05, ∗∗p ≤ 0.01, ∗∗∗p ≤ 0.001). (D) Erythrosine B inhibits FC-induced S6K1 phosphorylation under nutrient-free conditions. Representative western blot as in (A), except that a culture sample was pretreated for 15 min with EB (30 μM) before FC addition (EB + FC). (E) Quantification from independent experiments as in (D). Values and bars as in B. Asterisks indicate significant difference assessed with the two-tailed *t*-test (∗p ≤ 0.05 ∗∗p ≤ 0.01, ns denotes for not significant).

We next reasoned that TORC1 activation in FC-treated cells might be promoted by increased uptake of other nutrients (e.g., ammonium, nitrate, and sulfate), because of reinforcement of the plasma membrane H^+^ gradient following H^+^-ATPase stimulation. Furthermore, the culture medium also contains auxin known to promote TOR activation (Li et al., 2017; Schepetilnikov et al., 2017). Its uptake in root plant cells is reported to increase when H^+^-ATPases are stimulated (Meier et al., 2020), suggesting that TOR activation upon FC addition could also result from higher auxin influx. To assess these views, BY-2 cells grown in MS medium for three days were transferred for 2 h to an auxin-free and nutrient-free medium, namely MES buffer (pH 5.8). This shift caused, as expected, a strong decrease in S6K1 phosphorylation (Figure 5A). The starved cells were then treated with FC, which caused significant acidification of the external medium (Figure 5C). This acidification was much weaker than in previous experiments (Figures 3A and 3F), most likely because the buffering capacity of MES is higher than that of MS medium (Figure S4). Remarkably, FC addition under these conditions also induced a net increase in S6K1 phosphorylation (Figures 5A and 5B). To further assess the role of H^+^-ATPases in this FC-elicited TORC1 activation, cells were incubated for 15 min with erythrosine B before FC addition, which expectedly reduced acidification of the external medium (Figure 5C). Under these conditions, TORC1 activation by FC was much reduced (Figures 5D and 5E). Addition of erythrosine B also reduced the residual S6K1 phosphorylation still detectable in the nutrient-starved cells not treated with FC (Figures 5D and 5E). In conclusion, stimulating the activity of plasma membrane H^+^-ATPases promotes TORC1 activation even when sucrose, other nutrients or auxin are unavailable in the external medium.

In the aforementioned experiment, the MES buffer to which cells have been transferred contained a single micronutrient, potassium ions, added as KOH (∼1.7 mM) to adjust the buffer pH to 5.8. We thus reasoned that TORC1 activation in FC-treated cells might possibly be triggered by a change of K^+^ transport across the plasma membrane. For instance, previous studies showed that increased H^+^ efflux upon FC-induced H^+^-ATPase stimulation enhances K^+^ influx to compensate for membrane hyperpolarization (Marré and Ballarin-Denti, 1985). Furthermore, other studies reported that FC increases the conductivity of specific K^+^ channels by promoting their interaction with 14-3-3s (Saponaro et al., 2017). However, in our experiments, when cells grown in MS medium were transferred to the same K^+^-containing MES buffer, intracellular K^+^ was reduced but did not markedly further change upon FC addition (Figure S5). We also repeated the same FC treatment using cells which have been resuspended into MES buffer without adding KOH (Figure S6). Intracellular K^+^ was also reduced in these cells. Yet, FC addition still induced an external acidification of the medium as well as net increase in S6K1 phosphorylation. Furthermore, this TORC1 activation did not correlate with any significant variation of intracellular K^+^, which remained low (Figure S6).

In conclusion, stimulating the activity of H^+^-ATPases via FC-mediated neutralization of their self-inhibitory C-terminal tails triggers TOR signaling even when cells are resuspended in a buffer devoid of any nutrient and auxin.

## Discussion

In fungal and plant cells, H^+^-ATPases constitute the most abundant class of plasma membrane proteins. These highly conserved enzymes consume a significant part of the cellular ATP to establish a H^+^ gradient which drives active uptake of ions and nutrients and provides the main component of the membrane potential (Duby and Boutry, 2009; Falhof et al., 2016; Portillo, 2000; Serrano, 1993). These H^+^-pumps also participate in controlling the intracellular pH (Cosse and Seidel, 2021; Kane, 2016), and studies in plants have shown that they also play an important role in development and cell growth (Duby and Boutry, 2009; Falhof et al., 2016). In particular, stimulation of plant H^+^-ATPase activity in the presence of auxin promotes cell elongation. This was historically explained by the acid growth theory, according to which H^+^ pumps promote cell wall loosening (Takahashi et al., 2012) by acidifying the extracellular environment. In a more recent study, external acidification via H^+^-ATPases, after their stimulation upon NH_4_^+^ uptake, was reported also to promote lateral root development via protonation of auxin, which facilitates diffusion of the phytohormone across the plasma membrane (Meier et al., 2020).

We have previously reported that the yeast plasma membrane H^+^-ATPase (Pma1), upon stimulation by H^+^ influx or increase in the cytosol, promotes TORC1 activation (Saliba et al., 2018). Furthermore, the tobacco (*N. plumbaginifolia*) PMA2 H^+^-ATPase can substitute for Pma1 in supporting this TORC1 activation in yeast cells. This ability is lost when PMA2 is truncated of its regulatory C-terminal tail, and this loss does not correlate with reduced activity of the H^+^ pump (Saliba et al., 2021). These observations raise the question of whether H^+^-ATPases in plant cells might also contribute to TOR signaling. In this study, we have explored this hypothesis by first isolating a tobacco BY-2 cell line suitable for detecting rapamycin-sensitive TORC1 activation by monitoring increased phosphorylation of the T449 residue in an overexpressed S6K1 kinase as previously done with *Arabidopsis* cell cultures (Van Leene et al., 2019). Our results indicate that this BY-2 cell line is suitable, at least, for analyzing TOR control by sucrose (Van Leene et al., 2019; Xiong et al., 2013) and abscisic acid (Wang et al., 2018). Additional work is needed to determine whether other environmental cues known to influence TOR in plants (e.g., auxin, nitrogen, and sulfur) (Dobrenel et al., 2016; Wu et al., 2019) also impact TOR in our isolated BY-2 cell lines as can be expected.

The main conclusion of this study is that TOR activity in BY-2 cells is strongly influenced by the activity of plasma membrane H^+^ ATPases. It is indeed reduced when the H^+^ pumps are inhibited by erythrosine B or vanadate and upregulated when the cells are treated with fusicoccin (FC), which increases H^+^-ATPase activity. Importantly, FC also promotes TOR activation in cells transferred to a sucrose-free medium or even to a MES buffer devoid of any nutrient and auxin. In the latter medium, furthermore, FC-induced TOR activation depends on H^+^-ATPase activity because it is impeded by erythrosine B. The important role of plant H^+^-ATPases in TOR control is also illustrated by our observation that FC impedes BY-2 cell propagation and that this effect can be countered by adding rapamycin. This suggests that hyperactive H^+^-ATPases activate TOR to an extent that can become detrimental. This situation is reminiscent of the slow growth typically displayed by yeast mutants with a hyperactive TORC1, especially under nutrient limiting conditions (Neklesa and Davis, 2009). Our results thus show that stimulating or inhibiting the activity of plasma membrane H^+^-ATPases markedly influences TOR activity in BY-2 cells. In nutrient-fed cells, this impact on TOR can obviously be explained by the important role of H^+^ pumps in supporting active uptake of sugar, nitrogen, and other nutrients. As H^+^-ATPase activation also promotes diffusion of auxin (Meier et al., 2020), which promotes TOR upregulation (Li et al., 2017; Schepetilnikov et al., 2017), TOR activation upon H^+^-ATPase stimulation in BY-2 cells might also be promoted by increased auxin transport. Yet, the ability of H^+^-ATPases to promote TOR activation in cells transferred to a simple buffer indicates that these enzymes also influence TOR via other mechanisms. Hence the question: how do H^+^-ATPases promote TOR activation under such conditions?

As the activity of H^+^-ATPases influences intracellular pH (Cosse and Seidel, 2021; Kane, 2016), one could argue that treating BY-2 cells with FC causes alkalinization of the cytosol, which might be a signal promoting TOR activation. Conversely, inhibiting the activity of H^+^ pumps could cause significant acidification of the cytosol, potentially inhibiting TOR. It is worth noting that the cytosol has a natural buffering capacity and that the vacuolar V-ATPase activity should at least partially compensate for potential pH changes in the cytosol (Cosse and Seidel, 2021; Kane, 2016). Furthermore, the physiological relevance of direct control of TOR by intracellular pH is not obvious. In yeast, TORC1 activation is observed both when active nutrient uptake or inhibition of the V-ATPase causes a limited acidification of the cytosol and when a protonophore is used to cause greater increase in cytosolic H^+^ (Brito et al., 2019; Saliba et al., 2018). Yet, this acidification of the cytosol is not sufficient to promote TORC1 activation. Also required for this activation is the Pma1 H^+^-ATPase, which is stimulated under these conditions (Saliba et al., 2018). We therefore predict that the positive effect of FC on TOR activity in BY-2 cells is likewise because of stimulation of H^+^-ATPase activity rather than to a change in cytosolic pH, which in this case should be an alkalinization.

Stimulation of H^+^-ATPase activity by FC in plant cells induces a marked hyperpolarization of the plasma membrane, limited by induced K^+^ uptake (Marré and Ballarin-Denti, 1985). One could thus consider that this membrane hyperpolarization might trigger TOR activation, for instance by promoting increased influx of external K^+^ or other cations. However, this seems unlikely because TOR was also activated upon FC addition to cells transferred to a MES buffer without any added cation. An alternative scenario is that plasma membrane hyperpolarization somehow alters association or activity of peripheral membrane proteins which control early steps of a putative signaling cascade culminating with TOR upregulation.

TOR activation in FC-treated BY-2 cells might also be explained by a potential more direct role of stimulated H^+^-ATPases in TOR signaling, possibly via their C-terminal tail. This view is inspired by the capability of *N. plumbaginifolia* PMA2 H^+^-ATPase expressed in yeast of promoting TORC1 activation upon H^+^ increase or influx, in a manner dependent on its C-terminal tail (Saliba et al., 2021). This region of PMA2 is an autoinhibitory domain whose effect is countered by phosphorylation of several residues including the penultimate Thr (Duby and Boutry, 2009; Falhof et al., 2016; Haruta et al., 2015). Phosphorylation of this residue promotes binding of 14-3-3 dimers, favoring formation of a complex made of six H^+^-ATPases and six 14-3-3 proteins (Kanczewska et al., 2005; Ottmann et al., 2007). Importantly, experiments in BY-2 cells have shown that phosphorylation of the penultimate Thr in PMA2 increases under acidic conditions (Bobik et al., 2010) and also after incubation with FC (Kanczewska et al., 2005). It is thus tempting to consider that, once phosphorylated, the C-terminal tail of PMA2 ensures a role other than self-inhibition of H^+^ pumping, namely promoting TOR activation. In other words plant H^+^-ATPases, once their C-terminal tail is phosphorylated and in addition to actively pumping H^+^ out of the cell, might function as transceptors signaling to TOR, possibly after their hexamerization via binding to 14-3-3s. The consequence of such a mechanism would be stimulation of TOR signaling when H^+^-ATPases are fully active. Furthermore, this putative TOR control pathway via activated H^+^-ATPases might be conserved in yeast and perhaps other fungi. This could explain why a plant H^+^-ATPase such as *N. plumbaginifolia* PMA2, when expressed in yeast, can promote TORC1 activation dependent on its C-terminal tail and on endogenous yeast 14-3-3s (Saliba et al., 2021).

Whatever the signals and mechanisms underlying H^+^-ATPase-dependent TOR activation in yeast and plant cells, the view that these enzymes play an active role in TOR control is attractive, in the light of previously established interconnections between intracellular pH, H^+^-ATPase activity, and control of cell growth. For instance, although the intrinsic activity and intracellular trafficking of the yeast Pma1 H^+^-ATPase are tightly regulated according to cytosolic H^+^ (Kane, 2016), independent studies have shown that intracellular pH is an important signal in controlling yeast cell growth (Dechant and Peter, 2014; Dechant et al., 2014; Orij et al., 2012). In plants, several factors influencing cell growth and TOR signaling also tightly control the activity of H^+^-ATPases via phosphorylation of their C-terminal tails (Falhof et al., 2016). For instance, stimulation of H^+^-ATPase activity via phosphorylation of the C-terminal tail is induced in response to auxin (Takahashi et al., 2012), known to promote TOR signaling via the ROP2 small GTPase (Li et al., 2017; Schepetilnikov et al., 2017). Auxin-mediated activation of H^+^-ATPases involves induced synthesis of SAUR proteins, which interact with and inhibit PP2C-D clade type 2C phosphatases mediating dephosphorylation of the penultimate Thr in H^+^-ATPases (Spartz et al., 2014). Furthermore, auxin also promotes phosphorylation of this penultimate Thr via the TMK kinases (Li et al., 2021; Lin et al., 2021). The resulting activation of H^+^-ATPases provokes extracellular acidification, which according to the acid growth theory promotes cell expansion via loosening the cell wall (Hager, 2003). Yet cell expansion likely also requires major anabolic activity to provide novel lipids, proteins, and cell wall components. It thus seems worth considering that auxin-induced cell expansion might also involve TOR activation (Li et al., 2017; Schepetilnikov et al., 2017). Furthermore, this activation might be at least partially mediated by H^+^-ATPases stimulated via both the SAUR proteins and TMK kinases. More generally, previous works have shown that practically all factors influencing plant growth (light, phytohormones, extracellular peptides, and stress conditions) tightly control H^+^-ATPase activity by regulating phosphorylation of the C-terminal tail (Falhof et al., 2016). As more and more of these growth factors are reported to control TOR signaling (Wu et al., 2019), it will be important in future studies to assess the contribution of H^+^-ATPases in TOR regulation in different contexts of increased cell growth. For instance, a recent study reported that TOR is rapidly reactivated in nitrogen-depleted *Arabidopsis* seedlings upon uptake of NO_3_^-^, NH_4_^+^, or multiple single amino acids. Importantly, this TOR activation still occurs when assimilation of NO_3_^-^ into nitrite or NH_4_^+^ into glutamine is hampered (Liu et al., 2021). This situation resembles the one described in yeast where early TOR activation upon nitrogen uptake was shown to be elicited by H^+^ influx coupled to transport, in a manner dependent on the plasma membrane H^+^-ATPase (Saliba et al., 2018).

In conclusion, our study suggests that plant plasma membrane H^+^-ATPases play a potentially important role in TOR activation in a manner that goes beyond their role in driving active nutrient uptake. Additional work is needed to elucidate the underlying molecular mechanisms, whose comparison with those operating in yeast will also be of great interest.

### Limitations of the study

Our study is focused on BY-2 cell lines in which the *Arabidopsis* S6K1-HA kinase is overproduced. Although TOR activity in these cells responds as expected to sucrose and abscisic acid, additional work is needed to determine whether H^+^-ATPases also control TOR activity *in planta*.

## STAR★Methods

### Key resources table

**Table:** 

REAGENT or RESOURCE	SOURCE	IDENTIFIER
**Antibodies**		

Polyclonal rabbit antibody against peptide containing phospho-Thr449 from *Arabidopsis thaliana* S6K1	Agrisera	Cat# AS132664
Monoclonal rat antibody against HA peptide	Roche	Cat#11867423001; RRID:AB_390918
Polyclonal rabbit antibody (W1C) against *Nicotiana plumbaginifolia* PMA2 H^+^-ATPase purified from yeast	(Lefebvre et al., 2004; Maudoux et al., 2000)	

**Bacterial and virus strains**		

*Agrobacterium tumefaciens* LBA4404 *virG*(N54D)	(van der Fits et al., 2000)	

**Chemicals, peptides, and recombinant proteins**		

Abscisic acid	Sigma-Aldrich	Cat#A1049
AZD-8055	Hölzel Biotech	Cat#CD0348-025
Erythrosine-B (C.I 45430)	Sigma-Aldrich	Cat#115936
Fusicoccin	Enzo Life Sciences	Cat#BML-EI334-0001
Rapamycin	LCLabs	Cat#R-5000
Sodium-Ortovanadate	Sigma-Aldrich	Cat#S6508
MES	Sigma-Aldrich	Cat#M3671

**Experimental models: Cell lines**		

*N. tabacum cv. BY-2* suspension cells	(Nagata et al., 1992)	
*N. tabacum cv. BY-2 35S::AtS6K-3HA* suspension cells	This paper	
*N. tabacum cv. BY-2 35S::AtS6KT449A-3HA* suspension cells	This paper	

**Recombinant DNA**		

Plasmid: 35S::AtS6K-3HA	(Van Leene et al., 2019)	
Plasmid: 35S::AtS6KT449A-3HA	(Van Leene et al., 2019)	

**Software and algorithms**		

Image Studio Lite v 5.2.5 software	LI-COR	https://www.licor.com/bio/image-studio-lite/
Prism Version 9.2.0 software	Graphpad	https://www.graphpad.com/

### Resource availability

#### Lead contact

Further information and requests for resources and reagents should be directed to and will be fulfilled by the lead contact, Prof. Bruno André (Bruno.Andre@ulb.be).

#### Materials availability

This study did not generate new unique reagents.

### Experimental model and subject details

#### Plant cells

*N. tabacum cv. BY-2* suspension cells (Nagata et al., 1992) were grown as previously described (Niczyj et al., 2016) at 25°C in the dark in liquid MS medium composed of 0.44% Murashige and Skoog salts (MP Biomedicals), 30 g/L sucrose, 0.2 g/L KH_2_PO4, 0.2 mg/L 2,4-D, 2.5 mg/L thiamine-HCl, 50 mg/L myo-inositol, pH 5.8 (KOH). For the transgenic lines, the medium was supplemented with 100 μg/mL kanamycin. Typical cultures were grown in 50 mL of medium in a 250 mL Erlenmeyer flask on a rotatory shaker at 90 rpm. In all experiments, BY-2 cells from a seven-day preculture were initially used to inoculate cultures in fresh MS medium which were then incubated for three days. BY-2 cells were also maintained as calli on solid MS medium (0.8% agar).

### Method details

#### Generation of transgenic BY-2 cell lines

The plasmids used to generate transgenic lines overexpressing AtS6K1 and AtS6K1(T449A) (Van Leene et al., 2019) were kindly provided by Prof. G. De Jaeger (Ghent University). Briefly, the plasmid included, under the control of the 35S promoter, the AtS6K1 gene fused to a sequence coding for a C-terminal triple HA tag. These plasmids were transferred into *Agrobacterium tumefaciens* LBA4404 virG(N54D) (van der Fits et al., 2000) by electroporation.

Transformation of the BY-2 cells was performed as described in Navarre and Chaumont (2022). Briefly, a four-day BY-2 suspension culture was mixed with variable volumes of *A. tumefaciens* culture (OD_600_ = 1.5) in MS medium supplemented with 0.18% glucose, 0.1 mM acetosyringone, pH 5.3 (KOH) for 48 h at 25°C, in the dark without shaking. Two milliliters of co-culture were then plated on solid MS medium supplemented with 500 μg/mL cefotaxime, 400 μg/mL carbenicillin, 100 μg/mL kanamycin. The plates were incubated for 3–6 weeks at 25°C in the dark until calli emerged. The transformed calli were transferred at least twice to solid medium and then transferred into liquid medium.

#### Protein extraction and immunoblotting

To monitor changes in S6K1 kinase Thr449 phosphorylation, a previously published protocol (Urban et al., 2007) was applied with minor modifications. BY-2 cell samples (60-100 mg fresh weight) were mixed with TCA (final concentration 6%) and left on ice for at least 30 min. The cells were then collected by centrifugation, washed twice with cold acetone, and dried in a SpeedVac. Cell pellets were resuspended in 200 μL of 25 mM Tris pH 6.8, 6 M urea, 1% SDS and then lysed in the presence of 200 μL glass beads using a bead beater (5 cycles for 45 s) with subsequent heating for 10 min at 37°C.

Proteins were separated by 7.5% SDS-polyacrylamide gel electrophoresis and transferred to polyvinylidene fluoride membranes in a trans-blot semi-dry system (Biorad). Directly after transfer, the blots were incubated for 20 min in saturation buffer (5% or 2.5% dried milk, 0.5% Tween 20, 20 mM Tris-HCl, 8% NaCl, pH 7.6 (TBS-T)) and immunoblotted overnight at 4°C with either a polyclonal phospho S6 kinase 1 (p-T449) antibody (1:750) for detection of S6K1-T449 phosphorylation or a monoclonal anti-HA antibody (1:5000) (Roche) for S6K-HA detection. The total amount of H^+^-ATPases was detected with a polyclonal antibody (W1 C) against *N. plumbaginifolia* PMA2 purified from yeast (Lefebvre et al., 2004; Maudoux et al., 2000). Afterwards the blot was washed and, incubated with secondary antibodies (anti-rabbit or anti-rat, conjugated to peroxidase) for 1 h in 2.5% milk TBS-T, washed, and subjected to chemiluminescence detection with a luminol-like substrate (Roche). The signals were recorded with an Amersham Imager 600 (GE Healthcare). When necessary, membranes were stripped by means of a brief incubation in a buffer containing 0.4 N NaOH, followed by extensive washes with water, and re-probed as described above. Transfer quality and total protein levels were monitored by Direct blue 71 (Sigma-Aldrich) staining of the membrane as described (Zeng et al., 2013).

#### Measurement of acidification of the external medium by *N. tabacum* BY-2 cells

The pH of the external medium was monitored with a pH electrode. Two-milliliter aliquots of cell cultures were filtered, and supernatants were collected in order to manually record the external pH at the indicated times. Variation of the external pH was calculated as the difference between the final and initial pH values, normalized according to the cell density of the culture (fresh weight in grams).

#### Effect of long-term fusicoccin treatment on cell propagation

Cultures of S6K1-HA lines in MS sucrose medium were inoculated with samples of seven-day precultures and grown for three days. Then the cells were collected by centrifugation, and after removal of the supernatant, they were diluted to 10 mg/mL in fresh MS medium. After three additional days of growth, the fresh weight (FW) in mg per mL was measured in triplicate and the cells were split into three Erlenmeyer flasks for the indicated treatments: control (mock) and 2.5 μM fusicoccin in the presence or absence of 10 μM rapamycin. After 24 h, FW per mL was measured again in triplicate and the increment in FW after 24 h was measured (in mg/ml).

#### Measurement of intracellular potassium concentrations

Cultures of S6K1-HA 11 line in MS sucrose medium was inoculated with sample of seven-day preculture and grown for three days. The cells were diluted to 10 mg FW/ml in fresh MS sucrose medium. After three additional days of growth, the cells were collected (control) or incubated for 2 h in a nutrient-free solution (5 mM MES adjusted to pH 5.8 with KOH or 5 mM MES). The starved cells were then treated with 5 μM fusicoccin or with the vehicle ethanol. BY-2 cells were collected at different time intervals by filtration, washed twice with a solution of equal osmolarity (1.1 M sorbitol, 20 mM MgCl_2_), resuspended in 10 mL of H_2_O, dried at 95°C overnight, and then placed in a desiccator for 24 h. The dry matter was mineralized by heating at 500°C overnight. The ash sample was subsequently dissolved in 10 mL 6.5% HNO_3_ for analysis on an ICAP 6500 spectrometer (Thermo Scientific).

### Quantification and statistical analysis

The intensity of immunoblot signals was quantified with Image Studio Lite Version 5.2.5 software. Relative S6K1 phosphorylation was determined by calculating S6K1-pT449/S6K1-HA signal intensity ratios normalized using the reference condition set at 1.

Indicated P-values calculated using the two-tailed Student *t*-test were used to distinguish statistically significant differences between mean values. The number of replicates is specified in each figure legend. Graphs were generated with Prism Version 9.2.0 software.

## Data Availability

Any additional information required to reanalyze the data reported in this paper is available from the lead contact upon request. No code is produced in this paper.
