# Supplementary material for: Plasma membrane H+-ATPases promote TORC1 activation in plant suspension cells
Source: iScience. 2022 Apr 11;25(5):104238. doi: 10.1016/j.isci.2022.104238 (PMC9046228; doi:10.1016/j.isci.2022.104238)
Supplement: Document S1. Figures S1–S6 [file mmc1.pdf]

iScience, Volume *25*

Supplemental information

Plasma membrane H^+-ATPases promote TORC1 activation in plant suspension cells

Cecilia Primo, Catherine Navarre, François Chaumont, and Bruno André

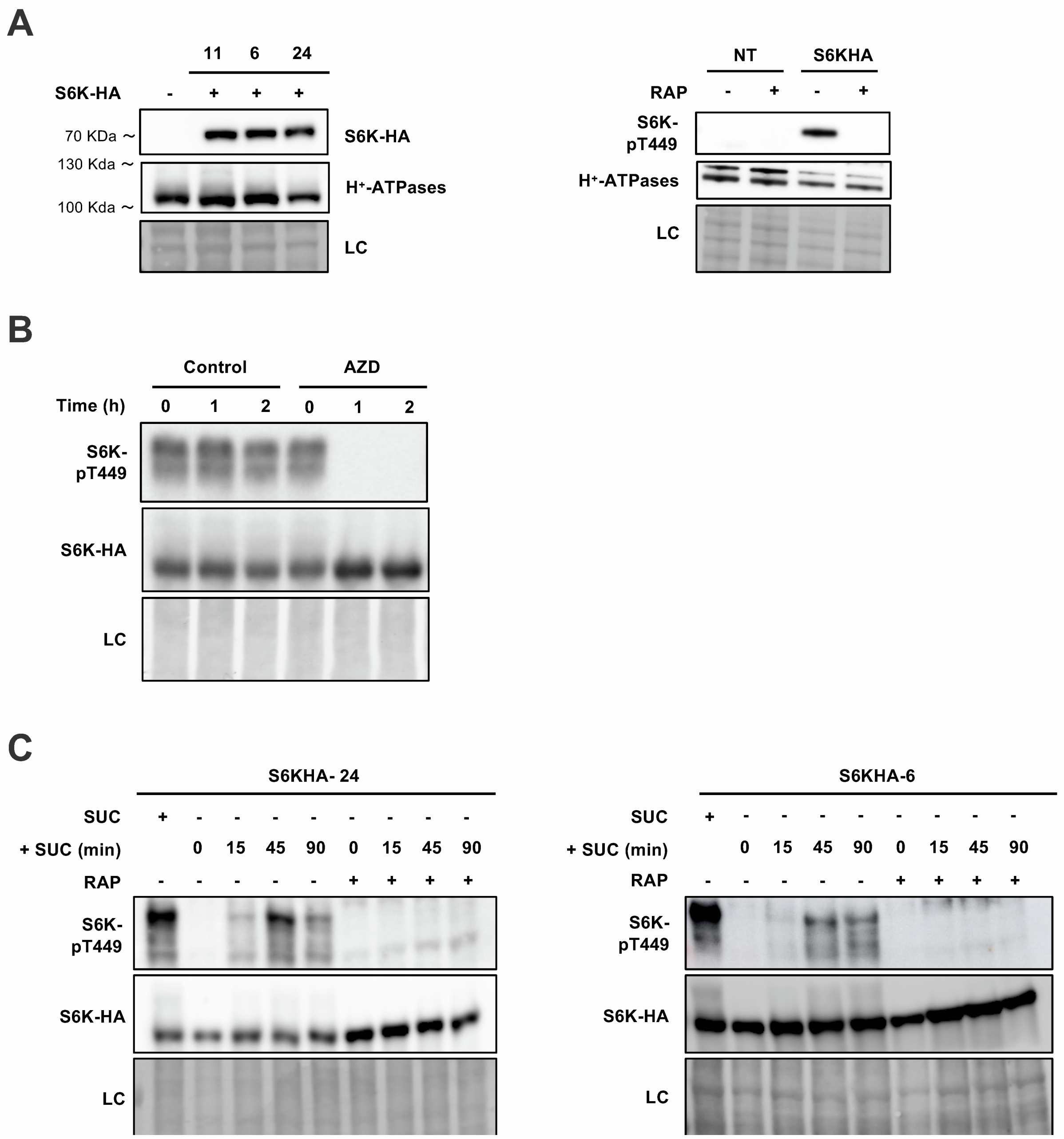

Figure S1. Isolation of different tobacco BY-2 cell lines suitable for studying TOR signaling, related to Figure 1

(A). (Left) Immunoblot illustrating similar expression of S6K1-HA in three stable BY-2 cell lines (+) isolated during this study (S6KHA-11, -6, and -24). The untransformed BY-2 cell line was used as a negative control (-). Cells pregrown in MS medium for three days were transferred to fresh medium for three more days. Protein extracts were prepared from cell samples and immunoblotted with antibodies against HA (upper panel) or H^+-ATPases (middle panel). The lower panel corresponds to the Direct blue-stained filter for detection of total proteins used as a loading control (LC). (Right) Immunoblot illustrating S6K-pT449 levels in the untransformed BY-2 cell line (NT) and a BY-2 cell line stably overexpressing S6K1-HA (S6KHA-

11). Cells grown in MS medium for three days were treated with 10 μM rapamycin (RAP) (+) or the vehicle DMSO. Protein extracts from cell samples were immunoblotted with anti-pT449-S6K1 (upper panel) and H^+-ATPases (middle panel) antibodies. The lower panel corresponds to a loading control (LC), as in (A).

(B). Representative immunoblot illustrating the effect of the specific ATP-competitive TOR inhibitor AZD8055 (AZD) on S6K1 phosphorylation in BY-2 cells. Cells expressing S6K1-HA (line S6KHA-11) were grown for three days in sucrose-containing MS medium, transferred to fresh MS medium for one more day, and treated for 0, 1 and 2 h with 1 μM AZD8055 (+) or the vehicle DMSO (-). Protein extracts prepared from cell samples were immunoblotted with anti-pT449-S6K1 (upper panel) and anti-HA (middle panel) antibodies. (C) Immunoblots as in Figure 1B except that cells were from the isolated BY-2 cell lines S6KHA-24 (left) and S6KHA-6 (right).

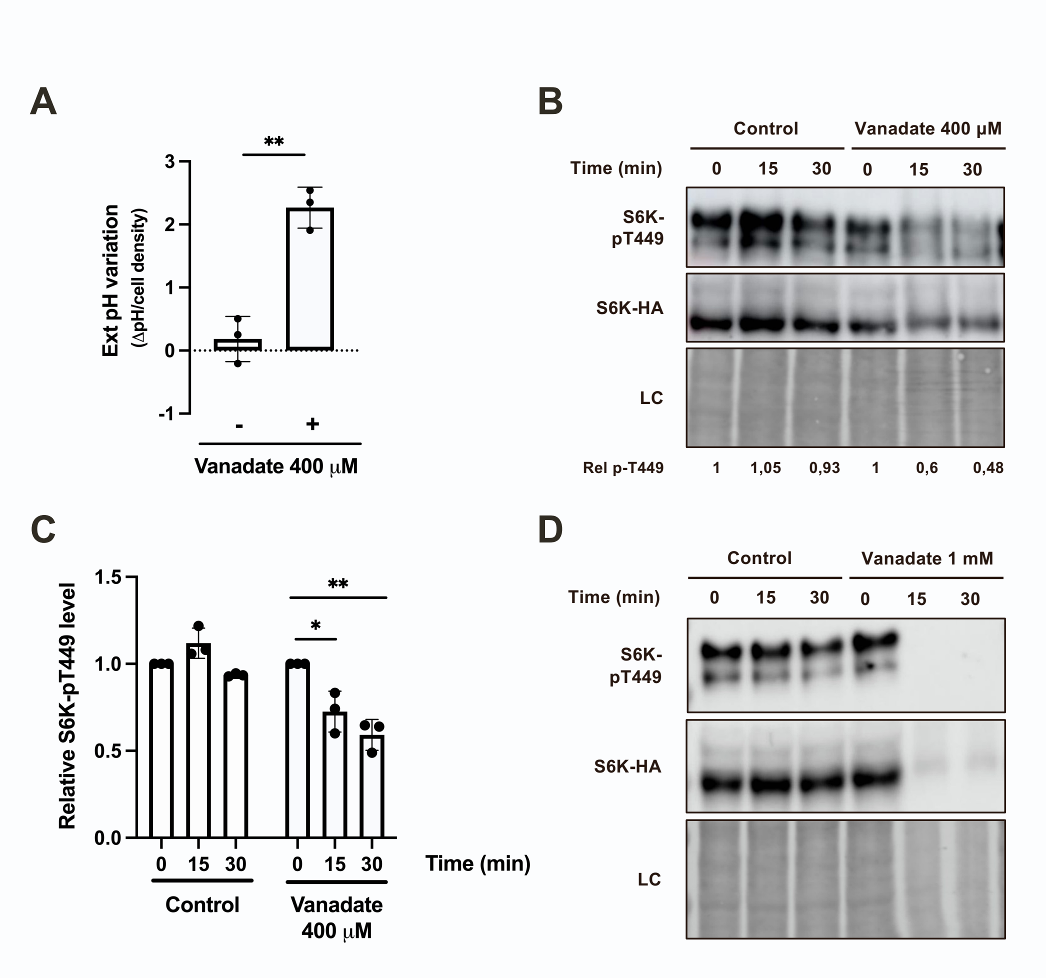

Figure S2. Inhibition of plasma membrane H+-ATPases by vanadate is associated with TOR inactivation, related to Figure 2

(A). External pH variation after cell treatment with vanadate. Cells of the BY-2 line S6KHA-11 pregrown in MS medium for three days and transferred to fresh medium for one more day were treated (+) or not (-) for 15 min with vanadate (400 μM). Bars represent means ± SD, n=3. Asterisks indicate significant differences assessed with a two-tailed *t*-test (**P <0.01).
(B). Representative immunoblot illustrating the influence of vanadate on S6K phosphorylation. Cells as in (A) were treated for the indicated times with vanadate (400 μM). Protein extracts from cell samples were immunoblotted with anti-pT449-S6K1 (upper panel) and anti-HA (middle panel) antibodies. The lower panel corresponds to a loading control (LC), as in Fig. S1. Rel p-T449 corresponds to relative S6K1-pT449 phosphorylation as detailed in C.
(C) Quantification, from independent experiments performed as in (B), of the influence of vanadate on S6K phosphorylation. Values represent S6K1-pT449 vs. S6K1-HA signal intensity ratios normalized using time '0 min' as the reference (set at 1), n=3. Bars represent means ± SD. Asterisks indicate significant differences assessed with a two-tailed *t*-test (*$P < 0.05$, **$P < 0.01$).
(D). Representative immunoblot as in (B) illustrating the influence of vanadate, added at a higher concentration (1 mM), on the total protein level (LC) (lower panel) as in Fig. S1A, the S6K-HA level (middle panel), and S6K1 T449 phosphorylation (upper panel).

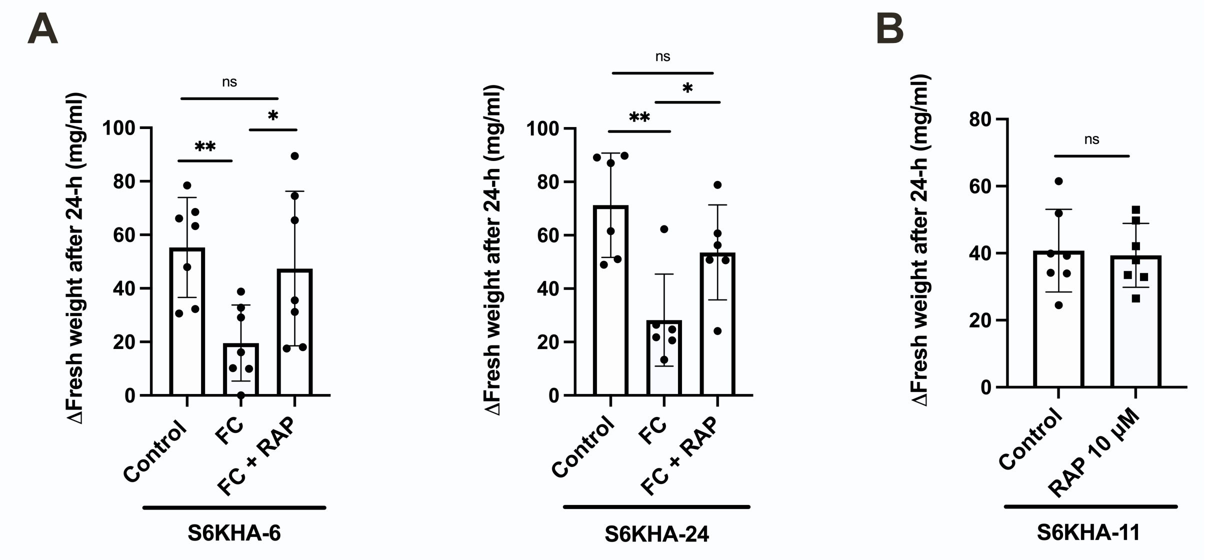

Figure S3. Stimulation of plasma membrane H⁺-ATPase activity by fusicoccin transiently activates TOR, related to Figure 3

(A) Influence of FC on growth. Cells of BY-2 lines S6KHA-6 and S6KHA-24 were pregrown in MS sucrose medium for three days and transferred to fresh medium for three more days. The cultures were then treated with FC (2.5 μM) in the presence or absence of rapamycin (10 μM), and the increase in fresh weight (mg/ml) was measured after 24 h. Control is untreated cells. Bars represent means ± SD (n≥6). Asterisks indicate significant differences assessed with the two-tailed *t*-test (**P* <0.05, ***P* <0.01, ns denotes not significant).
(B). Influence of rapamycin on growth. Cells of the BY-2 line S6KH1-11 cultivated as in (A) were treated with rapamycin (10 μM) and growth was measured after 24 h was as in (A). Bars represent means ± SD (n=7). Asterisks indicate significant differences assessed with a two-tailed *t*-test (ns denotes not significant).

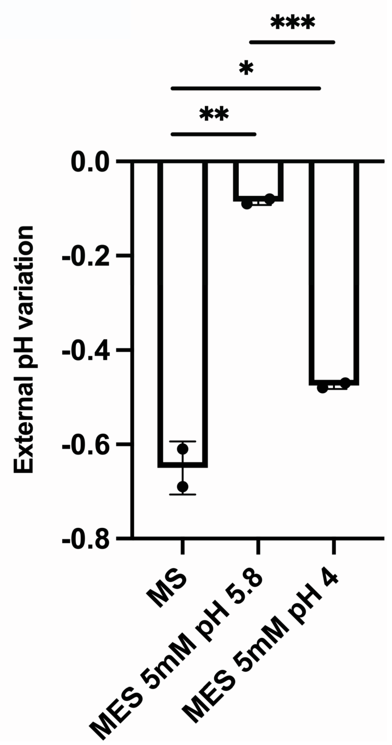

Figure S4. Comparison of the buffering capacities of MS medium and MES buffers used in fusicoccin-treatment experiments, related to Figure 5

pH change after addition of HCl (0,15 mM) to MS medium, MES buffer (5 mM) whose pH has been adjusted to 5.8 with KOH, and the same buffer whose pH has not been adjusted (measured pH: 4). Bars represent means ± standard deviation (n = 2). Asterisks indicate significant differences assessed with the two-tailed *t*-test (*P <0.05 **P <0.01, ***P <0.001).

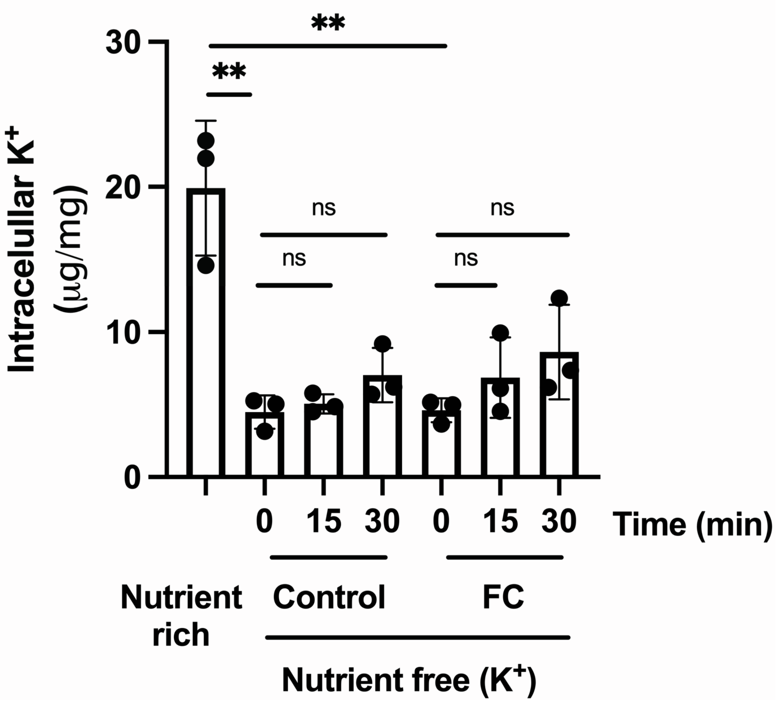

Figure S5. Influence of fusicoccin on intracellular K^+, related to Figure 5

BY-2 cells of S6KHA-11 line grown in MS medium for three days and transferred for three more days to fresh MS medium (“Nutrient rich”) were collected and transferred for 2 h to buffer referred as “Nutrient free” (5 mM MES, pH adjusted to 5.8 with KOH). The cells were then treated with FC (5 µM) or the vehicle ethanol (Control). Samples were taken at different time intervals and treated to measure the intracellular potassium concentration (n=3). Bars represent means ± SD. Asterisks indicate significant differences assessed with a two-tailed *t*-test (***P* < 0.01).

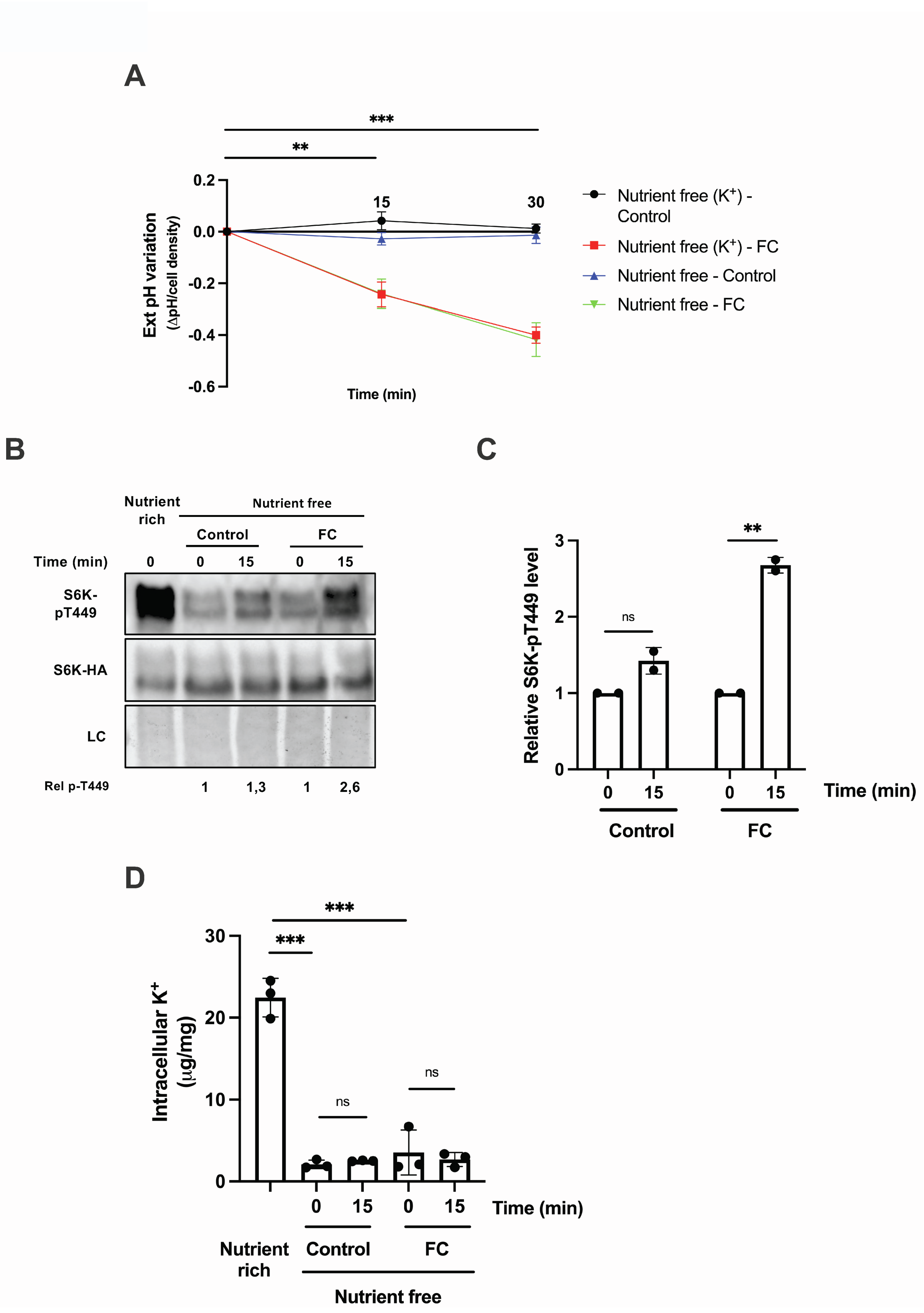

Figure S6. Stimulation of plasma membrane H$^+$-ATPase activity by fusicoccin transiently activates TOR under nutrient- and K$^+$-free conditions, related to Figure 5

(A). External pH variation after treating cells with fusicoccin (FC) under nutrient and K^+-free conditions. BY-2 cells of S6KHA-11 line grown in MS medium for three days and transferred for three more days to fresh MS medium were collected and transferred for 2 h to buffer referred as "Nutrient free (K^+)" (5 mM MES, pH adjusted to 5.8 with KOH) or "Nutrient free" (5 mM MES, unadjusted pH 4). The cells were then treated with FC (5 μM) or the vehicle ethanol (Control). Values are means ± SD, n=3. Asterisks indicate significant differences assessed with the two-tailed *t*-test (**P* < 0.05).

(B). Representative immunoblot illustrating the influence of fusicoccin (FC) on S6K1 phosphorylation under nutrient- and K^+-free conditions. Cells as in (A) were treated for 15 min with FC 5 μM or with the vehicle ethanol (Control). Protein extracts from cell samples were immunoblotted with anti-pT449-S6K1 (upper panel) and anti-HA (middle panel) antibodies. The lower panel corresponds to a loading control (LC), as in Fig. 1. As a positive control of S6K1 phosphorylation, a sample was collected before the shift to MES buffer (Nutrient rich, time 0). Rel p-T449 corresponds to relative S6K1-pT449 phosphorylation as detailed in Fig. 2C.

(C). Quantification, from independent experiments performed as in (A), of the influence of FC on S6K1 phosphorylation under nutrient and K^+-free conditions. Values represent S6K1-pT449 *vs.* S6K1-HA signal intensity ratios normalized using time '0 min' as the reference (set at 1), n=2. Bars represent means ± SD. Asterisks indicate significant differences assessed with the two-tailed *t*-test (**P* <0.05, ***P* <0.01, ****P* <0.001).

(D). Influence of fusicoccin (FC) on intracellular K^+ under under nutrient- and K^+-free conditions. Cells as in (A) were treated for 15 min with FC 5 μM or with the vehicle ethanol (Control) (n=3). Bars represent means ± SD. Asterisks indicate significant differences assessed with a two-tailed *t*-test (****P* <0.001).
